# CP690,550 inhibits oncostatin M-induced JAK/STAT signaling pathway in rheumatoid synoviocytes

**DOI:** 10.1186/ar3333

**Published:** 2011-05-06

**Authors:** Kiyoshi Migita, Atsumasa Komori, Takafumi Torigoshi, Yumi Maeda, Yasumori Izumi, Yuka Jiuchi, Taiichiro Miyashita, Minoru Nakamura, Satoru Motokawa, Hiromi Ishibashi

**Affiliations:** 1 Department of Rheumatology and Clinical Research Center, Nagasaki Medical Center, Kubara 2-1001-1, Omura 856-8652, Japan

## Abstract

**Introduction:**

Interleukin (IL)-6-type cytokines exert their effects through activation of the Janus kinase/signal transducers and activators of transcription (JAK/STAT) signaling cascade. The JAK/STAT pathways play an important role in rheumatoid arthritis, since JAK inhibitors have exhibited dramatic effects on rheumatoid arthritis (RA) in clinical trials. In this study, we investigated the molecular effects of a small molecule JAK inhibitor, CP690,550 on the JAK/STAT signaling pathways and examined the role of JAK kinases in rheumatoid synovitis.

**Methods:**

Fibroblast-like synoviocytes (FLS) were isolated from RA patients and stimulated with recombinant oncostatin M (OSM). The cellular supernatants were analyzed using cytokine protein chips. IL-6 mRNA and protein expression were analyzed by real-time PCR method and ELISA, respectively. Protein phosphorylation of rheumatoid synoviocytes was assessed by Western blot using phospho-specific antibodies.

**Results:**

OSM was found to be a potent inducer of IL-6 in FLS. OSM stimulation elicited rapid phosphorylation of STATs suggesting activation of the JAK/STAT pathway in FLS. CP690,550 pretreatment completely abrogated the OSM-induced production of IL-6, as well as OSM-induced JAK/STAT, and activation of mitogen-activated kinases (MAPKs) in FLS.

**Conclusions:**

These findings suggest that IL-6-type cytokines contribute to rheumatoid synovitis through activation of the JAK/STAT pathway in rheumatoid synoviocytes. Inhibition of these pro-inflammatory signaling pathways by CP690,550 could be important in the treatment of RA.

## Introduction

Rheumatoid arthritis (RA) is a chronic inflammatory disease that is characterized by the activation and proliferation of synovial tissues with associated degradation of articular cartilage [1]. Synovial fibroblasts are believed to play an important role in rheumatoid synovitis through the production of a variety of inflammatory mediators [2]. Activation of synovial fibroblasts is mediated in large part by cytokines, such as IL-1 or TNF-α, which are produced by monocytes/macrophages [3]. However, other cytokines likely participate in the process of synovial cell activation. Of the IL-6-related cytokines, oncostatin M (OSM) is another product of macrophages and activated T cells that is elevated in the synovial fluids of RA patients [4,5]. Moreover, OSM stimulates chemokine and matrix metalloproteimase (MMPs) production suggesting its important effects in synovial inflammation [6]. IL-6-type cytokines exert their effects via the signal transducer gp130 leading to the activation of the Janus kinase (JAK)/signal transducer and activator of transcription (STAT) cascade [7]. In brief, the ligand-receptor interaction elicits the assembly of cytokine receptors, receptor-associated JAKs, which recruit and activate STAT proteins. Phosphorylated STATs then dimerize, translocate to the nucleus and direct transcription of the target genes [8]. Recently, JAK inhibition has been shown to have a prominent effect on autoimmune diseases [9]. CP690,550 is an orally available JAK antagonist that is in development for the treatment of RA and other autoimmune conditions [10,11]. Furthermore, a recent clinical trial demonstrated that CP690,550 is efficacious in RA, resulting in rapid, significant reductions in the signs and symptoms of RA [12,13]. The role of oncostatin M in diseases is less well defined, but recent studies suggest that it might be involved in inflammatory cell recruitment and cartilage destruction in RA [14]. In the present study, we used primary human rheumatoid synoviocytes and demonstrated the induction of multiple signaling cascades and a critical role of the JAK/STAT pathway in the oncostatin M-mediated IL-6 synthesis. Furthermore, we showed that interference of the JAK/STAT pathway using CP690,550, a JAK kinase inhibitor, completely abrogated the OSM-induced IL-6 production in rheumatoid synoviocytes.

## Materials and methods

### Patients

All RA patients fulfilled the American College of Rheumatology criteria for RA [15]. Synovial tissue samples were obtained from seven patients with RA during synovectomy. The whole study was approved by the Ethics Committee of the Nagasaki Medical Center and informed consent was obtained from each of the individuals.

### Reagents

JAK inhibitor CP690,550 was obtained from Axon Biochemicals (Postbus, Netherlands). Human recombinant OSM was purchased from R&D Systems (Minneapolis, MN, USA). Human recombinant IL-6 and soluble IL-6 receptor (sIL-6R) were purchased from Peprotech (Rocky Hills, NJ, USA). PD98059, SB203580, SP600125 and pyridone 6 (2-*tert*-butyl-9-fluoro-3,6-dihydro-7*H*-benz [*h*]-imidaz (4,5-*f*) isoquinoline-7-one) were obtained from Calbiochem (San Diego, CA, USA). Phospho-specific and pan antibodies against JAK-1 (Tyr1022/1023), JAK-2 (Tyr1007/1008), STAT-1 (Tyr701), STAT-3 (Tyr705), STAT-5 (Tyr694), ERK-1/2 (Thr202/Tyr204), p38 (Thr180/Tyr182), c-Jun N-terminal kinase (JNK; Thr183/Tyr185) and β-actin were purchased from Cell Signaling Technology (Beverly, MA, USA). Phospho-specific and pan antibodies against JAK3 (Tyr980) were purchased from Santa Cruz Biotechnology (Santa Cruz, CA, USA).

### Preparation of FLS

Synovial tissue was obtained from patients with RA at the time of total joint replacement or synovectomy. Synovium was minced and incubated with 1 mg/ml collagenase type VIII (Sigma-Aldrich, St. Louis, MO, USA) in serum-free RPMI 1640 medium (Life Technologies, Grand Island, NY, USA) for one hour at 37°C, filtered, extensively washed, and cultured in DMEM (Life Technologies) supplemented with 10% FBS in a humidified 5% CO_2 _atmosphere. FLS were used from passages 4 through 5 during which time they were a homogeneous population of cells (<1% CD 45 positive).

### Cytokine and chemokine assays in synoviocytes-conditioned medium

The levels of cytokines and chemokines in FLS-conditioned media were measured using a RayBio Human Cytokine Antibody Array 5 (Ray Biotech, Inc. Norcross, GA, USA), according to the manufacturer's instructions. This assay employs a qualitative Western screening technique. The standard array matrix consisted of an 11 × 8 dot grid on a 20 mm × 30 mm nitrocellulose membrane with 79 unique capture antibodies. The array kit included the biotinylated-antibodies solution and chemiluminescent substrate. The cytokine array membrane was incubated with 1 ml of rheumatoid synoviocyte-conditioned media for two hours, and the membrane was then washed three times with washing buffer 1 for five minutes each, followed by washing buffer II for five minutes each. Cytokines were detected using cytokine antibody for one hour, followed by HRP-labeled strepavidin incubation for one hour. The dilution used for each of these reagents was indicated by the instructions contained in the assay kit. Reactive spots were visualized by enhanced chemiluminescence (ECL) (Amersham Pharmacia Biotech UK Limited, Little Chalfont, UK) with exposure to X-ray film.

### Measurement of cytokine secretion

FLS (5 × 10^4^) were seeded in 24-well plates containing RPMI plus 10% FCS for 24 hours. Following 24 hours of incubation in serum-free RPMI, the cells were stimulated with OSM for 24 hours. Cell-free supernatants were collected by centrifugation at 400 g for five minutes and assayed for IL-6 with enzyme-linked immunosorbent assay (ELISA) kits (R&D Systems, Minneapolis, MN, USA) according to the manufacturer's instructions.

### Reverse transcription-polymerase chain reaction (RT-PCR)

Total RNA was extracted from FLS using the RNeasy total RNA isolation protocol (Qiagen, Crauley, UK). cDNA was prepared with Superscript reverse transcriptase (Invitrogen, Grand Island, NY, USA). The amplification of the IL-6 transcripts was accomplished on a Light Cycler (Roche Diagnostics, Mannheim, Germany) using specific primers. The housekeeping gene fragment of glyceraldehydes-3-phosphates dehydrogenase (GAPDH) was used for verification of equal loading.

### Cell lysis and Western blotting

Serum-starved FLS were stimulated for 20 min with OSM indicated in the figure legends and the cells were washed by ice-cold PBS and lysed with a lysis buffer (1% Nonidet P 40, 50 mM Tris, pH 7.5, 100 mM NaCl, 50 mM NaF, 5 mM EDTA, 20 mM β-glycerophosphate, 1.0 mM sodium orthovanadate, 10 μg/mL aprotinin and 10 μg/mL leupeptin) for 20 minutes at 4°C. Insoluble material was removed by centrifugation at 15,000 × g for 15 minutes at 4°C. The supernatant was saved and the protein concentration was determined using the Bio-Rad protein assay kit (Bio-Rad, Hercules, CA, USA). An identical amount of protein (50 μg) for each lysate was subjected to 10% SDS-polyacrylamide gel electrophoresis, and then transferred to a nitrocellulose membrane. Western blot analysis using phospho-specific anti-JAKs, STATs and MAPKs antibodies was performed with an ECL Western blotting kit (Amersham, Little Chalfont, UK).

### Statistical analysis

All quantitative data are presented as the mean ± SD of independent experiments using different FLSs. Statistical comparison between treatments was performed using one-way analysis of variance (ANOVA) and *post hoc *Tukey's test. *P-*values less than 0.05 were considered statistically significance.

## Results

### OSM induced IL6 secretion by rheumatoid synoviocytes

**Figure 1:**
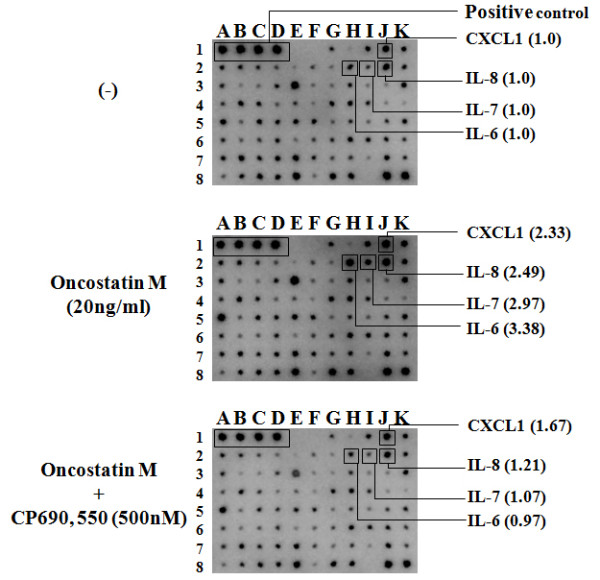
**OSM induces IL-6 synthesis from RA-FLS**. RA-FLSwere stimulated with recombinant OSM (20 ng/day) in the presence or absence of CP690,550 (500 nM) for 24 hours. The levels of cytokines and chemokines in RA-FLS-conditioned media were subjected to cytokine antibody array as described in Material and methods. The indicated dots of cytokines or chemokines were calculated as a relative unit by densitometer. The dots of each protein of untreated condition (upper column) were assigned the value of 1.0 and the density of each dots of OSM or OSM plus CP690,550 treated condition (middle or lower column) was expressed as the relative unit. Two experiments were performed using different RA-FLS and a representative result is shown.

**Figure 2:**
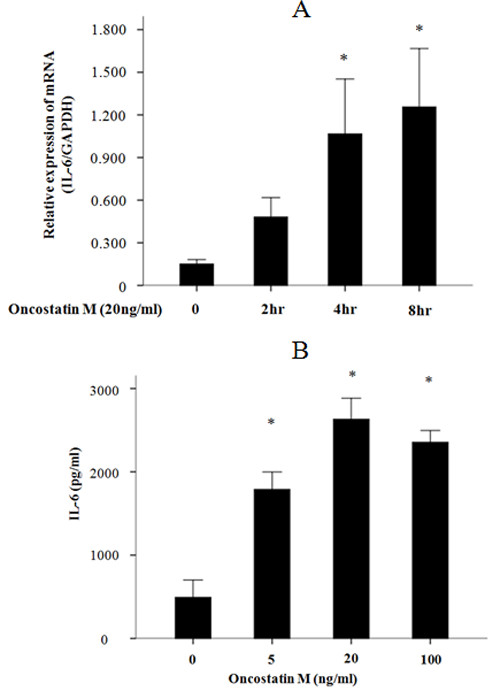
**OSM stimulus IL-6 mRNA expression in RA-FLS, OSM stimulates IL-6 synthesis in RA-FLS. (a) **RA-FLS were stimulated with 20 ng/ml of recombinant OSM for various times as indicated. IL-6 and GAPDH mRNA expression was determined by real-time PCR method. The data were expressed as the mean ± SD of three independent experiments. *P < 0.01 compared to untreated RA-FLS. (**b) **RA-FLS were stimulated with various concentrations of recombinant OSM as indicated for 24 hours. IL-6 protein in the conditioned media was determined by ELISA. The data were expressed as the mean ± SD of three independent experiments. *P < 0.01 compared to untreated RA-FLS.

**Figure 3:**
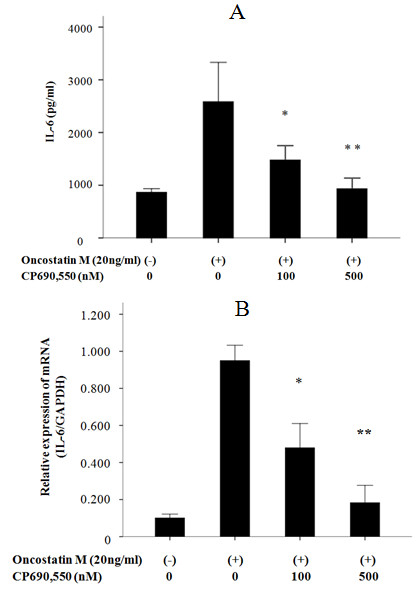
**CP690,550 suppresses OSM-induced IL-6 synthesis in RA-FLS; CP690,550 suppresses OSM-induced IL-6 mRNA expression in RA-FLS. (a) **RA-FLS were pretreated with vehicle (DMSO, -) or indicated concentrations of CP690,550 for two hours. Cells were stimulated with OSM (20 ng/ml) for 24 hours, after which conditioned media were collected and IL-6 content was measured by ELISA. The data were expressed as the mean ± SD of three independent experiments. *P < 0.05 compared to OSM-treated RA-FLS. **P < 0.01 compared to OSM-treated RA-FLS. **(b) **RA-FLS were pretreated with vehicle (DMSO, -) or indicated concentrations of CP690,550 for two hours. Cells were stimulated with OSM (20 ng/ml) for four hours, after which, IL-6 and GAPDH mRNA expression was determined by real-time PCR method. The data were expressed as the mean ± SD of three independent experiments. *P < 0.01 compared to OSM-treated RA-FLS. **P < 0.001 compared to OSM-treated RA-FLS.

We initially examined whether OSM stimulates cytokine and chemokine production by FLS. The human cytokine/chemokine protein chip was used to analyze the changes in the induction of cytokine and chemokine in FLS after stimulation with OSM. The culture supernatants from OSM-stimulated FLS in the presence or absence of CP690,550 were subjected to cytokine/chemokine microarray. Data from a representative experiment are depicted in Figure 1. OSM stimulated the cytokines or chemokines production from RA-FLS. Among these, we focused on the cytokine, which was highly induced by OSM and suppressed by CP690,550. OSM stimulated the induction of IL-6, which was prevented in the presence of CP690,550. Verification of the array data by real-time PCR and ELISA analyses confirmed that OSM promotes IL-6 mRNA and protein expression in FLS. The responsiveness of FLS to OSM was evaluated by stimulating the cells with OSM. OSM increased IL-6 mRNA expression (Figure 2A) and IL-6 protein synthesis (Figure 2B) in RA-FLS. The minimal dose of 20 ng/ml of OSM was sufficient to induce IL-6 protein synthesis. CP690,550 pretreatment abolished the OSM-mediated IL-6 induction in a dose-dependent manner (Figure 3).

### OSM induces activation of the JAK/STAT pathways

**Figure 4:**
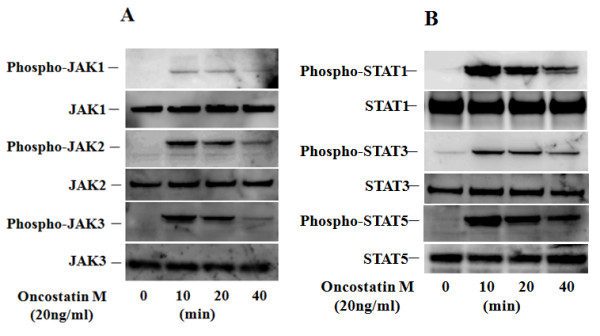
**Phosphorylation of JAKs/STATs in OSM-treated RA-FLS**. Quiescent RA-FLS were stimulated with OSM (20 ng/ml) for indicated times. Phosphorylation of JAKs (A; JAK1, JAK2, JAK3) and STATs (B; STAT1, STAT3, STAT5) were determined by Western blotting using phospho-specific or pan antibodies against JAK1, JAK2, JAK3, STAT1, STAT3 and STAT5. Three experiments were performed using different RA-FLS and a representative result is shown.

**Figure 5:**
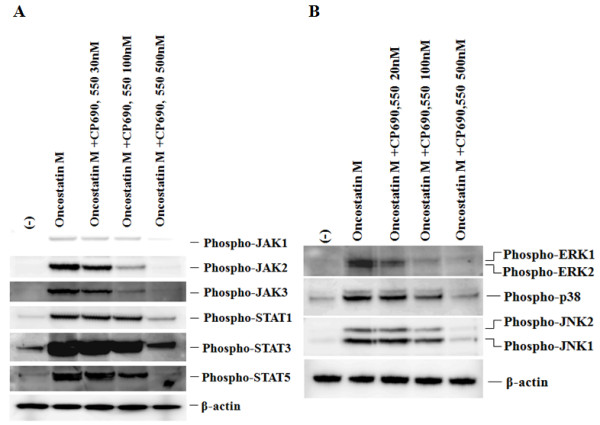
**CP690,550 suppresses OSM-induced JAKs/STATs activation in RA-FLS; CP690,550 inhibits OSM-induced MAPKs activation RA-FLS**. **(a) **Quiescent RA-FLS were pretreated with various concentrations of CP690,550 for 2 hours, then stimulated with OSM (20 ng/ml) for 20 minutes. Cellular lysates were subjected to Western blotting using phospho-specific antibodies against JAK1, JAK2, JAK3, STAT1, STAT3 and STAT5. Three experiments were performed using different RA-FLS and a representative result is shown. **(b) **Quiescent RA-FLS were pretreated with various concentrations of CP690,550 for 2 hours, then stimulated with OSM (20 ng/ml) for 20 minutes. Cellular lysates were subjected to Western blotting using phospho-specific antibodies against ERK1/2, p38 and JNK1/2. Three experiments were performed using different RA-FLS and a representative result is shown.

To investigate the mechanisms of OSM-mediated signaling in FLS, we evaluated the activation of JAKs and STATs in OSM-treated synoviocytes. Quiescent FLS were stimulated with 20 ng/ml of OSM for different time periods (0 to 40 minutes), and protein extracts were analyzed by immunoblotting with phosphospecific antibodies. OSM-stimulated phosphorylation of JAK1, JAK2, JAK3, STAT1, STAT3 and STAT5, occurred within 10 minutes and peaked at 20 minutes (Figure 4). CP690,550 blocked the OSM-induced phosphorylation of the JAKs and STATs in a dose-dependent manner (Figure 5A).

### Effects of CP690,550 on the MAPK pathways

To investigate the possible interaction between the JAK and MAPKs pathways, FLS were pretreated with CP690,550, stimulated with OSM and protein extracts were analyzed using phospho-specific anti-MAPKs antibodies. OSM stimulated phosphorylation of ERK1/2, p38 and JNK1/2 and CP690,550 attenuated the phosphorylation of these MAPKs in a dose-dependent manner (Figure 5B).

### p38 pathway inhibition suppresses OSM-induced IL-6 production

**Figure 6:**
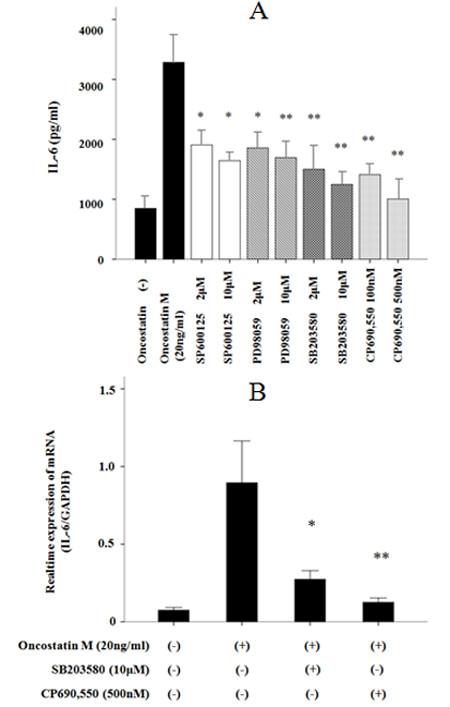
**p38 inhibition suppresses OSM-induced IL-6 induction; p38 inhibition suppresses OSM-induced IL-6 mRNA expression**. **(a) **RA-FLS pretreated with vehicle (DMSO-), PD98059 (ERK1/2 pathway inhibitor), SB203580 (p38 inhibitor), and SP600125 (JNK inhibitor) for two hours. Cells were then stimulated with OSM (20 ng/ml) for 24 hours, after which conditioned media were collected and IL-6 content was measured by ELISA. The data were expressed as the mean ± SD of three independent experiments. *P < 0.01 compared to OSM-treated RA-FLS. **P < 0.001 compared to OSM-treated RA-FLS. **(b) **RA-FLS pretreated with vehicle (DMSO-) or SB203580 (p38 inhibitor), for two hours. Cells were then stimulated with OSM (20 ng/ml) for four hours, after which IL-6 and GAPDH mRNA expression was determined by real-time PCR method. The data were expressed as the mean ± SD of two independent experiments. *P < 0.05 compared to OSM-treated RA-FLS. **P < 0.01 compared to OSM-treated RA-FLS.

We examined whether MAPK activation was functionally linked to the OSM-induced production of IL-6. To investigate the role of MAPKs in OSM induction of IL-6 expression, FLS were pre-treated with inhibitors specific for each of the MAPKs, ERK1/2, p38 and JNK1/2, and then stimulated with OSM. As shown in Figure 6A, inhibition of JNK or ERK1/2 signaling led to partial reduction of the OSM-induced IL-6 secretion without affecting cellular viability which was checked by trypan blue exclusion test showing that >95% of cells that were treated with each inhibitors exclude trypan blue. Meanwhile, inhibition of p38 resulted in an almost complete abrogation of IL-6 secretion. To determine whether p38 inhibitors affect the expression of IL-6 mRNA, we examined IL-6 mRNA expression using real-time PCR methods. SB203580, a p38 specific inhibitor dose-dependently reduced the OSM-stimulated IL-6 mRNA induction in FLS (Figure 6B).

### Pyridone 6 inhibits OSM-induced JAK/STAT activation

**Figure 7:**
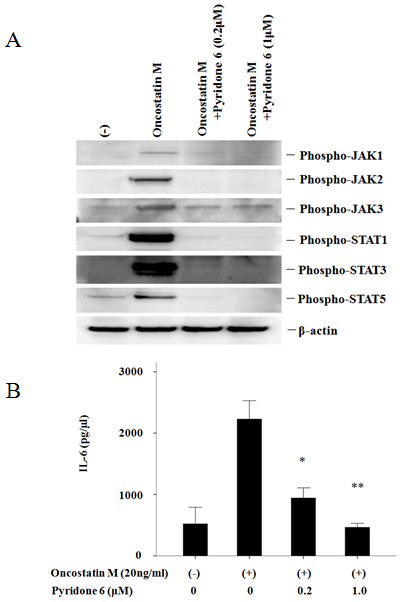
**Pyridone 6 suppresses OSM-induced JAKs/STATs activation in RA-FLS; Pyridone 6 suppresses OSM-induced IL-6 synthesis in RA-FLS**. **(a) **Quiescent RA-FLS were pretreated with vehicle (DMSO -) or pyridone 6 for 2 hours, then stimulated with OSM (20 ng/ml) for 20 minutes. Cellular lysates were subjected to Western blotting using phospho-specific antibodies against JAK1, JAK2, JAK3, STAT1, STAT3 and STAT5. Two experiments were performed using different RA-FLS and a representative result is shown. **(b) **Quiescent RA-FLS were pretreated with vehicle (DMSO, -) or pyridone 6 for two hours, then stimulated with OSM (20 ng/ml) for 24 hours. IL-6 protein in the conditioned media was determined by ELISA. The data were expressed as the mean ± SD of two independent experiments. *P < 0.05 compared to OSM-treated RA-FLS. **P < 0.01 compared to OSM-treated RA-FLS.

**Figure 8:**
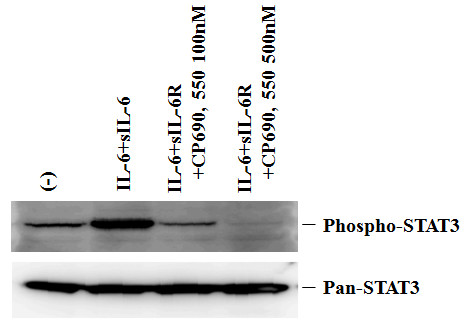
**CP690,550 suppresses IL6-induced STAT3 activation in RA-FLS**. Quiescent RA-FLS were stimulated with IL-6 (100 ng/ml) plus sIL-6R (100 ng/ml) for 20 minutes. Phosphorylation of STAT3 were determined by Western blotting using phospho-specific or pan antibodies against STAT3. Three experiments were performed using different RA-FLS and a representative result is shown.

To confirm these effects induced by JAK inhibitor, CP690,550, on RA-FLS, we used a different compound that blocks JAK. We examined the effects of a pan-JAK inhibitor, pyridone 6 [16], on OSM-mediated signaling in RA-FLS. As shown in Figure 7A, pyridone 6 suppressed OSM-induced JAK (1, 2, 3) and STAT (1, 3, 5) activation in RA-FLS. Also, pyridone 6 inhibited OSM-induced IL-6 production from RA-FLS (Figure 7B). Finally, we examined whether CP690,550 affects IL-6 receptor (IL-6R) pathway, which activates STAT3 through gp130. IL-6 plus sIL-6R induced STAT3 activation in RA-FLS. CP690,550 almost completely inhibited STAT3 activation induced by IL-6 plus sIL-6R in RA-FLS (Figure 8).

## Discussion

In this study, we demonstrated that OSM activates the JAK/STAT and MAPK pathways in FLS. Furthermore, CP690,550 blocked the OSM-induced STAT phosphorylation, probably by affecting upstream JAKs. These results strongly suggest that the JAK/STAT pathways are essential for OSM-mediated rheumatoid inflammatory responses, and can be blocked by CP690,550. The IL-6 type cytokine OSM is known to be involved in the pathogenesis of RA [4]. It is secreted by activated T cells, monocytes, and neutrophils, and elevated levels of OSM are detected in the synovial fluid of patients with RA but not OA [4,5]. OSM is known to activate the JAK/STAT signaling cascade in other systems [17], but this has not been properly elucidated in rheumatoid synoviocytes. Our demonstration of OSM-triggered phosphorylation of JAKs/STATs in primary rheumatoid synoviocytes suggests that these cells could be a source of activated STATs in rheumatoid synovial tissues [18]. CP690,550 is an orally available JAK antagonist that is currently in development for the treatment of RA and other autoimmune conditions [11-13]. Blockade of OSM-induced phosphorylation of JAK1, JAK2 and JAK3 by CP690,550 suggests that this JAK inhibitor also affected JAK1 and JAK2, in addition to JAK3. This notion was further supported by the abolishment of downstream STAT1, STAT3 and STAT5 activation and OSM-induced IL-6 production in rheumatoid synoviocytes. These results suggest the pivotal role of the JAK/STAT pathway in OSM signaling leading to rheumatoid inflammatory responses.

The biological agents targeted against tumor necrosis factor-α (TNF-α) have been shown to successfully suppress rheumatoid inflammation and have been thought to be more efficacious than disease-modifying anti-rheumatic drugs (DMARDs) in controlling joint damage [19]. The combination of DMARDs with biological agents can increase the response rate; however, about 30% of patients do not respond to initial treatment [20]. This has prompted research into alternative methods to suppress rheumatoid disease activity. JAK3 is critical for signal transduction from the common γ-chain of the receptors for IL-2, IL-4, IL-7, IL-9, IL-15 and IL-21 on the plasma membrane to the nuclei of immune cells [21]. These cytokines bind to cytokine receptors and signal through the JAK3-signal transducer and activator of transcription (STAT) pathways [22]. Therefore, a better understanding of JAK/STAT activation in the rheumatoid synovium may allow the development of a novel therapeutic strategy. Agents that selectively inhibit JAK3 have the potential to mediate potent immune modulation, affecting lymphocytes, macrophages and NK cells [23,24]. CP690,550 was originally believed to be a JAK3 inhibitor. However, it is now clear that in addition to its effect on JAK3, this compound inhibits JAK1 and JAK2 at similar concentrations [25]. Interestingly, CP690,550 was shown to be effective in preventing joint damage in collagen-induced arthritis (CIA), an animal model of rheumatoid arthritis [26]. In CIA, IL-6 is a critical cytokine to induce arthritis [27]. In this study, we have demonstrated that CP690,550 inhibited OSM-induced IL-6 production from rheumatoid synoviocytes by affecting JAK/STAT signaling. The exact mechanism by which CP690,550 prevents CIA remains to be determined, it is possible that CP690,550 inhibits IL-6 induction as well as IL-6-mediated signaling by affecting the JAK/STAT pathway. We have provided evidence that CP690,550 is a potent inhibitor of the JAK/STAT pathway with *in vitro *activity in rheumatoid synoviocytes consistent with *in vitro *enzyme assay. Although the signaling pathways for IL-6 induction by proinflammatory cytokines and stimuli have been reported in many cell types [28], no data have been available on the regulation of IL-6 by OSM-signaling pathway. Our data clearly demonstrated that JAK/STAT activation play a pivotal role in OSM-mediated IL-6 up-regulation in RA. IL-6 is considered to be a critical cytokine that drives inflammatory joint destruction in RA [28]. Furthermore, the targeting of IL-6 has been shown to induce a therapeutic benefit in RA [29]. CP690,550 could be useful in blocking the JAK/STAT-mediated proinflammatory responses including IL-6-mediated signaling in RA.

The MAPKs play an important role in the induction of pro-inflammatory cytokines in rheumatoid synovitis [30]. From the experiments using the specific MAPKs inhibitors, we concluded that OSM-mediated IL-6 release is weakly dependent on the ERK1/2 or JNK signaling, but depended mostly on the p38 signaling pathway. Our findings are consistent with previous studies showing the essential role of p38 in IL-6 production from activated rheumatoid synoviocytes [31]. Dimerization of IL-6 type cytokine receptors does not only lead to activation of the JAK/STAT-signaling pathway, but also to the induction of MAPK [17]. The relationship between the JAK/STAT pathway and the Ras/MAPK pathway is complex and these pathways cross at multiple levels [32]. The molecular mechanism linking JAK/STAT engagement to the activation of MAPKs remains to be analyzed in detail. Our findings provide the possibility that p38 MAPK may be partly involved in the JAK/STAT-mediated IL-6 induction.

JAK1 and JAK2 are more widely expressed, whereas JAK3 expression is mostly limited to haematopietic cell lines [33]. Although JAK3 has been identified as a potential therapeutic target in autoimmune disease, its role in rheumatoid synovitis has not been fully elucidated. In this study, we clearly demonstrated the JAK-family including JAK3 could be activated in FLS in response to an IL-6-type cytokine, OSM. Our data suggest that cytokine-activated JAKs in FLS could be an appropriate therapeutic target in inflammatory arthritis including RA.

## Conclusions

We demonstrated that OSM activates the JAK/STAT signaling pathway in RA-FLS followed by MAPK activation leading to the induction of IL-6. CP690,550 blocked OSM-induced JAK/STAT activation, as well as MAPK resulting in the abrogation of OSM-responsive induction of IL-6. Therefore, the JAK/STAT pathways are essential in the transduction of OSM signaling and regulation of IL-6 genes inRA-FLS. Modulation of the JAK/STAT pathways by CP690,550 represents an alternative therapeutic strategy to regulate rheumatoid inflammation.

## Abbreviations

CIA: collagen-induced arthritis; DMARDs: disease-modifying anti-rheumatic drugs; ECL: enhanced chemiluminescence; FCS: fetal calf serum; FLS: fibroblast-like synoviocyte; GAPDH: glyceraldehydes-3-phosphates dehydrogenase; IL: interleukin; JAK: Janus kinase; MAPKs: mitogen-activated kinases; MMPs: matrix metalloproteimase; OSM: oncostatin M; STAT: signal transducers and activators of transcription; RA: rheumatoid arthritis; TNF-α: tumor necrosis factor-alpha.

## Competing interests

The authors declare that they have no competing interests.

## Authors' contributions

KM, AK, TT, YM and YJ carried out cell culture and biochemical analysis. YI, TM and MN participated in the design of the study and performed the statistical analysis. SM and HI conceived the study, participated in its design and coordination and helped to draft the manuscript. All authors read and approved the final manuscript.
